# Therapies for IDH-Mutant Gliomas

**DOI:** 10.1007/s11910-023-01265-3

**Published:** 2023-04-15

**Authors:** Ruham Alshiekh Nasany, Macarena Ines de la Fuente

**Affiliations:** 1 Upstate University Hospital and Cancer Center, Syracuse, NY USA; 2 Sylvester Comprehensive Cancer Center and Department of Neurology, 1120 NW 14th Street, Miami, FL 33136 USA

**Keywords:** Glioma, Oligodendroglioma, Astrocytoma, *IDH* mutations, *IDH* inhibitors, Immunotherapy

## Abstract

**Purpose of Review:**

Isocitrate dehydrogenase (*IDH*) mutant gliomas are a distinct type of primary brain tumors with unique characteristics, behavior, and disease outcomes. This article provides a review of standard of care treatment options and innovative, therapeutic approaches that are currently under investigation for these tumors.

**Recent Findings:**

Extensive pre-clinical data and a variety of clinical studies support targeting *IDH* mutations in glioma using different mechanisms, which include direct inhibition and immunotherapies that target metabolic and epigenomic vulnerabilities caused by these mutations.

**Summary:**

*IDH* mutations have been recognized as an oncogenic driver in gliomas for more than a decade and as a positive prognostic factor influencing the research for new therapeutic methods including IDH inhibitors, DNA repair inhibitors, and immunotherapy.

## Introduction

Gliomas are the most common malignant primary brain tumors in adults [1, 2]. Classification of these tumors has evolved in recent years with the identification of molecular features including isocitrate dehydrogenase (*IDH*) mutations in gliomas in 2008 [3]. In humans, the IDH family includes three isoforms: IDH1, IDH2, and IDH3. All three forms are essential for several metabolic processes, such as the Krebs cycle. Recognized as an oncogenic event [4–6], *IDH* mutations are highly prevalent in gliomas and confer significant improved survival when compared to the *IDH* wild-type (*IDH*-WT) glioma [7–9].

The World Health Organization (WHO) Classification of Central Nervous System (CNS) Tumors (2021) classifies adult-type diffuse glioma based on the presence of *IDH* mutations and other key molecular alterations in astrocytoma *IDH* mutant, oligodendroglioma *IDH* mutant and 1p/19q-co-deleted, and glioblastoma *IDH*-WT [10].

Standard of care therapy for *IDH* mutant gliomas starts with maximal safe resection when feasible. Surgery has both diagnostic and therapeutic objectives and, in the majority of the cases, is followed by a combination of radiation and chemotherapy. The radiation dose depends on the grade of glioma, and the chemotherapy relies on a variety of factors including, but not limited to, histopathological categories, age at diagnosis, comorbidities, and physician preference [11–13].

Although significant advances have been made in the molecular characterization of gliomas, clinicians face challenges in managing this disease. Standard of care options offer limited survival benefits and may result in long-term toxicities including cognitive decline. This affects quality of life and remains a significant burden for patients and their families [14]. This article provides an overview of *IDH* mutant gliomas, standard of care treatment, and novel therapeutic strategies, including *IDH* inhibitors, agents targeting DNA repair mechanisms and epigenetic vulnerabilities, and immunotherapy.

### Molecular Aspects and Classification

Historically, glioma classification relied on histology and immunohistochemistry to describe the tumors’ microscopic appearance and classify a spectrum of tumors with overlapping features. However, in 2016 the World Health Organization (WHO) introduced molecular markers in its classification for the first time, allowing these tumors to be defined by their molecular features resulting in a more accurate classification and prognosis [15–18]. These changes were expanded in the 2021 WHO Classification of CNS Tumors [10].

**Fig. 1:**
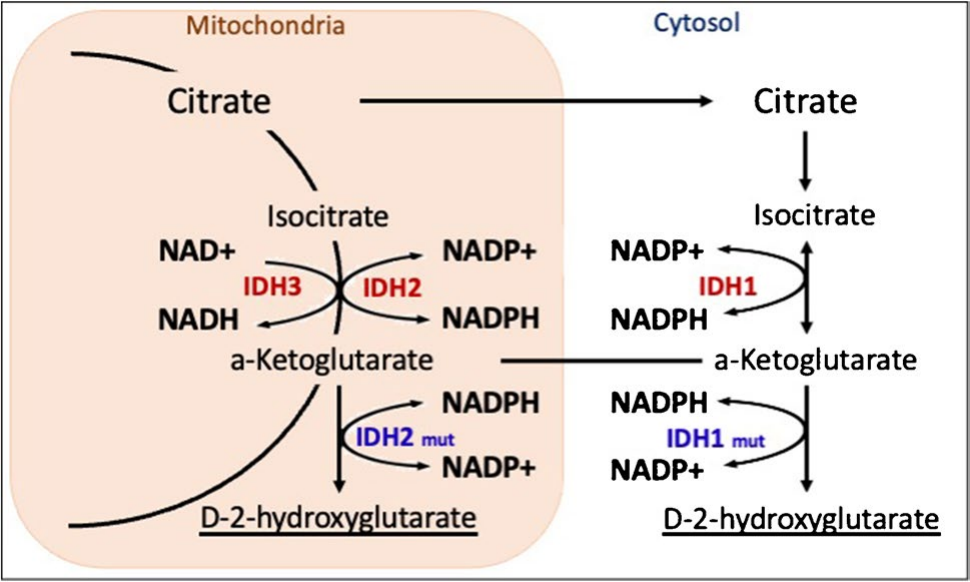
The *IDH* enzymes family and their role in cell metabolism as well as the effect of *IDH* mutations on the Krebs cycle and the accumulation of D-2-HG

One of the key molecular alterations incorporated into the WHO Classifications of CNS Tumors in 2016 are mutations in IDH enzymes, which were originally discovered in 2006 in colorectal cancer and reported in gliomas in 2008 [3, 19, 20]. The IDH enzyme family consists of three isoforms that catalyze the oxidative decarboxylation of isocitrate to produce α-ketoglutarate. *IDH* mutations, occurring early in glioma oncogenesis, lead to the accumulation of the oncometabolite D-2-hydroxyglutarate (2-HG), which is linked to metabolic and epigenetic dysregulation and includes inhibition of normal cellular differentiation and hypermethylation that leads to disease [21–23] (Fig. 1).

**Fig. 2:**
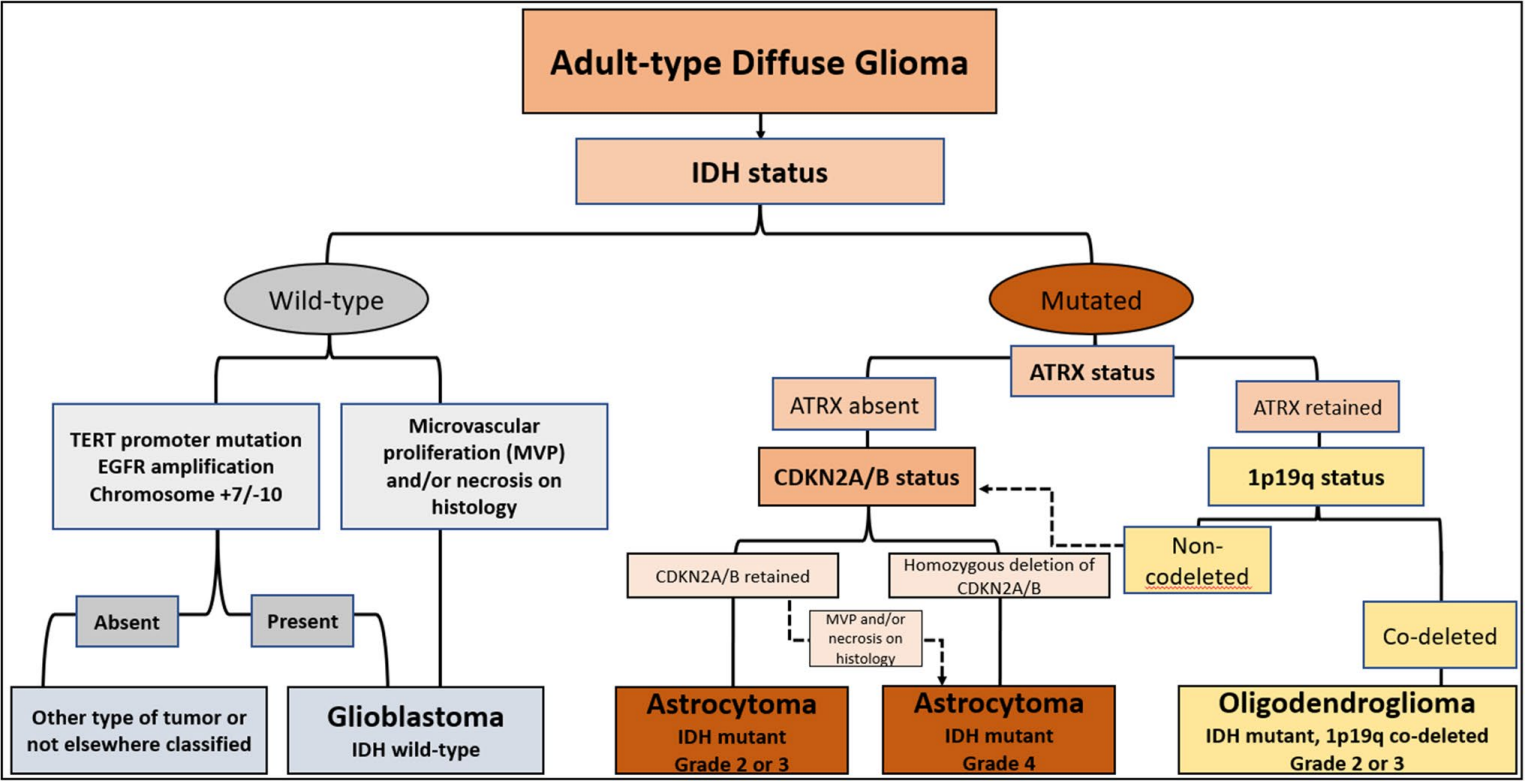
The 2021 WHO classification of adult-type diffuse gliomas

The 2021 WHO classification includes two adult-type diffuse *IDH* mutant gliomas, oligodendrogliomas (grades 2 or 3) and astrocytomas (grades 2, 3, or 4). The system also classifies tumors that do not carry *IDH* mutations (*IDH*-wildtype) in the presence of other molecular features, such as Epidermal growth factor receptor (EGFR) amplification, TERT promoter mutation, or the gain of chromosome seven with loss of chromosome 10 (+7/-10) to grade 4 glioblastomas regardless of their histologic appearance [10]. The 2021 WHO classification also defines oligodendrogliomas by the loss of chromosomes 1p and 19q (1p19q co-deletion) compared to astrocytomas that do not carry that feature (Fig. 2). Classifying *IDH* mutant tumors into grades 2 and 3 in oligodendrogliomas and 2, 3, and 4 in astrocytomas continues to rely on histologic appearance, except when astrocytic tumors carry homozygous deletion of CDKN2A/B making them grade 4 tumors independent of histologic characteristics. [24].

### Clinical Manifestation and Prognosis

Patients with gliomas harboring *IDH* mutations typically present at a younger age compared to *IDH* wild type gliomas, with a peak incidence between ages of 35 and 44, according to the Central Brain Tumor Registry of the United States (CBTRUS) data base report. *IDH* mutant gliomas have a higher incidence rate in males with an approximate male to female ratio of 1.3 [25, 26]. Low-grade *IDH* mutant gliomas have a variety of presenting symptoms. Reports show that up to 80% of the patients have seizures as an initial symptom [13, 27]. Less frequently, patients present with focal neurological deficits, cognitive and behavioral changes, and signs of increased intracranial pressure. These tumors are often found incidentally with brain imaging while patients are undergoing evaluation for unrelated symptoms or during trauma assessments [28, 29]. Conversely, high-grade gliomas harboring *IDH* mutations may present more aggressively with a faster clinical deterioration [30].

*IDH* mutant gliomas appear as expansive lesions on magnetic resonance images (MRI) with hyperintensity on T2-weighted images and T2-fluid-attenuated inversion recovery sequences (FLAIR). Grade 2 *IDH* mutant gliomas do not commonly enhance upon the administration of IV gadolinium, unlike grade 3 and 4 *IDH* mutant tumors that often enhance or have an enhancing component within a larger non-enhancing tumor. *IDH* mutations are prognostic factors for longer survival independent of the histologic phenotype, and they improve progression free survival (PFS) [5, 6, 9, 31]. Moreover, *IDH* mutations are associated with better outcomes in high-grade gliomas as well. The median overall survival for patients with *IDH mutant* astrocytoma, CNS WHO grade 3 (previously known as anaplastic astrocytoma), is 65 months versus 20 months for *IDH* wild type [32, 33].

### Standard of Care Therapies

In discussing therapy modalities for *IDH* mutant gliomas, this review differentiates between low-grade gliomas carrying these mutations as grade 2 oligodendrogliomas and astrocytomas versus *IDH* mutant high-grade gliomas, which include grade 3 oligodendrogliomas and grades 3 and 4 astrocytomas. This acknowledges the different behavior, and subsequently, the different management modalities between these low- and high-grade gliomas.

### Surgery

Surgery remains the most important first step for glioma diagnosis and management. Historically, surgery was performed on patients presenting with large or symptomatic lesions, or when the initial imaging revealed features of high-grade pathology, such as the presence of edema, enhancement, or necrosis [34, 35]. The timing of surgery in small and non-enhancing tumors had been debated, especially in tumors that are asymptomatic and found incidentally; however, recently the field has moved towards performing early surgical resection [36].

A Norwegian study published in 2017 retrospectively analyzed the cases of 153 patients in two centers with different surgical treatment strategies. The first center favored early surgical resection of low-grade tumors, and the other favored observation following a diagnostic biopsy. The researchers found that the overall survival (OS) of patients treated at the center that favored early surgical resection was 14.4 years (95% CI 10.4–18.5) compared to 5.8 years (95% CI 4.5–7.2) in the center that favored observation (*p*=0.001) [37]. Multiple other retrospective and observational studies confirmed improved survival with early surgical resection, even for the small and asymptomatic tumor [38, 39]. In addition, early surgical intervention allows for a definitive pathologic and molecular diagnosis that informs treatment selection and determines prognosis.

Clinical experts agree on the benefit of surgical resection compared to a needle biopsy for an accurate diagnosis given the heterogeneity of gliomas [40, 41]. From the therapeutic perspective, the extent of surgical resection has a significant effect on the clinical outcomes of both high- and low-grade gliomas. In non-enhancing *IDH* mutant gliomas, extensive resection that targets all the non-enhancing disease area remains the standard first-line strategy. For enhancing tumors, the goal is to resect all of the enhancing component. Many of the studies supporting surgical resection were conducted prior to the molecular characterization of these tumors [42, 43]. However, modern era studies confirm these resection strategies impact clinical outcomes. One recent study retrospectively evaluated 228 tumors histologically classified as low-grade gliomas. The researchers molecularly re-evaluated these tumors to update their classification based on the 2016 WHO classification and found that the residual post-operative volume negatively affected OS with a hazard ratio of 1.01 (95% CI: 1.002–1.02; *P* = 0.016) per cm3 increase in volume [44]. Certain studies advocate for supramaximal resection of gliomas beyond the non-enhancing disease component and include a prospective study with 449 patients by Rossi et al. [45, 46]. However, the extension of the resection needed outside the border of visible tumor to achieve better outcomes is still unclear and long-term survival data is still needed [47]. The benefit of extensive surgical resection also applies to high-grade gliomas harboring *IDH* mutations. Multiple studies have shown that extensive resections of both enhancing and non-enhancing tumor components are associated with significant survival benefit [48, 49].

### Radiation Therapy

Radiation therapy (RT) continues to play a significant role in managing *IDH* mutant gliomas; however, there are long-term side effects on cognition. To address this, researchers are evaluating the timing of RT during treatment course. More specifically, they are studying the safety effects of delaying RT in patients with “low-risk” features defined as younger than 40 years old, gross total resection of their tumor, positive for 1p-19q co-deletion, and good performance status and neurological function [50–52].

The timing of post-operative radiation in low grade *IDH* mutant gliomas has been controversial, but there is growing evidence for delaying radiation in certain cases. The EORTC 22845, a study by the European Organization for Research and Treatment of Cancer, was conducted in the pre-*IDH* era before gliomas were classified by *IDH* mutations. The study compared immediate radiation versus salvage radiation at progression in histologically-defined grade 2 gliomas. Results published in 2005 reported no difference between the two treatments in OS [53]. A study by the Radiation Therapy Oncology Group (RTOG 9802) followed low-risk grade 2 glioma patients who were defined as younger than 40 years old, had a gross total resection of their tumor, good performance status and neurological function, and received delayed radiation at progression. In addition, serial MRI observation did not adversely affect OS in these patients [52]. Based on this evidence, low-risk patients with *IDH* mutant low-grade gliomas can be observed with serial MRI scans, and radiation can be deferred till progression.

However, for the treatment of high-risk grade 2 glioma patients and patients with grade 3 and 4 gliomas harboring *IDH* mutations, immediate radiation post-operatively is recommended [26, 54].

The modality of radiation is also being assessed for impact on long-term side effects by comparing proton and photon therapy. A prospective single-arm study observed 20 patients with grade 2 gliomas, who were treated with proton therapy, and followed them over a median of 5.1 years. Results revealed no decline in the patients’ quality of life or cognitive deficits [55]. An on-going phase II clinical trial by the NRG Oncology Group (previously RTOG) is comparing proton versus photon radiation therapy in *IDH* mutant grade 2 or 3 gliomas.

### Chemotherapy

Studies in the late 1990s and early 2000s began reporting a response in low-grade gliomas using chemotherapy alkylating agents, such as temozolomide and regimens of procarbazine, lomustine, and vincristine (PCV) [56–60]. The RTOG 9802 protocol (discussed above) included another phase II study for high-risk patients with grade 2 gliomas [57]. The study defined these high-risk patients as age 40 or older and those who had a sub-total resection or biopsies of their tumors. The study reported tumor regression in a meaningful proportion of patients using PCV. Based on these results, a phase III trial took place randomizing patients with high-risk grade 2 glioma to radiation alone versus radiation followed by six cycles of PCV. Patients treated with radiation alone had a median OS of 7.8 years versus 13.3 years in the radiation plus PCV arm (HR, 0.59; *p* = .003) [52]. Because these initial studies took place prior to the molecular era, the researchers in RTOG 9802 later performed a subset analysis and found that the improvement in overall median survival was statistically significant in patients with tumors harboring *IDH1* mutations. Bell et al. assessed the same tumors from RTOG 9802 that had sufficient tissue with genomic sequencing to identify *IDH* mutation and 1p/19q co-deletion. The survival improvement seen in the original analysis was reported in patients with tumors harboring these mutations and not in the *IDH* wild-type [61].

Similar observations were seen with RTOG 9402 and EORTC 26951 studies that looked at patients with *IDH* mutant high-grade gliomas and tumors formerly known as anaplastic oligodendrogliomas (grade 3). In these randomized studies, patients received radiation alone versus radiation and PCV. Both trials reported improvement in OS in the PCV arm, which was limited to the tumors harboring 1p/19q co-deletion [62, 63].

Moreover, the addition of chemotherapy improves outcomes in high-grade gliomas with *IDH* mutations and intact 1p/19q confirming the predictive value of *IDH* mutations independently from the 1p/19q co-deletion status. The CATNON trial looked at grade 3 gliomas with *IDH* mutations and intact 1p/19q. Seven hundred fifty-one patients were randomly assigned (1:1:1:1) to radiotherapy alone, radiotherapy with concurrent oral temozolomide, radiotherapy with adjuvant oral temozolomide (12 cycles), or radiotherapy with both concurrent and adjuvant temozolomide. Adjuvant temozolomide chemotherapy, but not concurrent temozolomide chemotherapy, was associated with a survival benefit in patients with 1p/19q non-co-deleted anaplastic glioma. The clinical benefit was dependent on *IDH1* and *IDH2* mutational status [64, 65].

The ongoing CODEL phase III trial evaluates newly-diagnosed patients with *IDH* mutant, 1p/19q co-deleted gliomas grades 2 and 3, and compares the use of radiation followed by PCV versus radiation with concurrent temozolomide followed by adjuvant temozolomide [66]. Although the efficacy between temozolomide and PCV is still unclear, guidelines, including those recently published by an expert panel of the American Society of Clinical Oncology (ASCO) and the Society for Neuro-Oncology (SNO), allow both treatments to be used [54, 67]. Temozolomide’s toxicity profile is more manageable, and physicians may consider patient’s age and co-morbidities among other factors when deciding on chemotherapy options [64].

Despite the above-mentioned advances in managing gliomas with *IDH* mutations, there is still no known curative therapy, and patients continue to suffer premature death and cognitive decline.

### Advances in Therapy Modalities

Without a curative therapy for *IDH* mutant gliomas regardless of the grade, research is ongoing for novel therapies. Several approaches are being investigated, including targeted therapies, immunotherapies, and other approaches.

### IDH Inhibitors

In 2013, researchers published pre-clinical data on the prototype mIDH1 inhibitor AGI-5198 reporting that it inhibited both biochemical and cellular production of D-2-HG. In vivo studies revealed that AG1-5198 impaired growth of *IDH* mutant glioma cells and induction of glial differentiation [68, 69]. However, AGI-5198 had poor pharmaceutical properties that prevented its further use in clinical trials.

Subsequently, ivosidenib (mIDH1 inhibitor) and enasidenib (mIDH2 inhibitor) were developed and tested in patients with hematologic malignancies with *IDH* mutations, such as acute myeloid leukemia (AML). The Food and Drug Administration (FDA) approved these treatments for that indication [70]. More recently, olutasidenib has also obtained regulatory approval in recurrent AML.

The role of IDH inhibitors in glioma treatment, however, is still under investigation. Two phase I studies evaluating ivosidenib (mIDH1 inhibitor) and vorasidenib (mIDH1/2 inhibitor), in 66 and 93 patients respectively, reported a benign safety profile. Results showed patients treated with ivosidenib had prolonged stable disease and reduced growth of the non-enhancing tumors, while vorasidenib showed an overall response rate of 18% in non-enhancing gliomas [71, 72]. Another recent phase 1b/2 study tested olutasidenib, a selective *mIDH1* inhibitor, on 26 patients with recurrent, *mIDH1* gliomas (mainly enhancing tumors) and reported tolerability as well as preliminary clinical activity in a heavily pre-treated group of patients [73]. Other mIDH inhibitors are currently under clinical investigation, such as BAY1436032, DS-1001, LY3410738, and more [74, 75].

Despite the mIDH inhibitors’ therapeutic promise, reports suggest potential limitations. Some studies found that while different IDH inhibitors reduced the accumulation of D-2-HG, they failed to reverse global DNA or histone hypermethylation responsible for oncogenesis [76, 77]. Other studies suggest that there is potential for resistance to DNA-damage-inducing therapies, such as radiation and chemotherapy [78, 79]. Additional clinical trials looking at the efficacy of IDH inhibitors in glioma are ongoing. These include a phase III trial studying vorasidenib versus a placebo in patients with residual or recurrent *IDH* mutant gliomas grades 2 or 3 (NCT04164901) and a study of DS-1001 in patients with chemotherapy- and radiotherapy-naïve *IDH1*-mutated WHO grade 2 gliomas (NCT04458272). These clinical studies may help elucidate the precise role of these therapies in cancer treatment and the optimal timing for therapy.

### DNA Methyltransferase Inhibitors (DNMTi)

As discussed earlier, *IDH* mutations lead to a hypermethylation phenotype that results in epigenetic alterations in glioma cells and is possibly linked to gliomagenesis [77, 80]. Correcting this epigenetic dysregulation was studied as a potential therapeutic strategy for *IDH* mutant gliomas. In 2013, two papers reported that 5-azacytidine and decitabine (both DNMTi agents) induced differentiation of glioma cells and reduced tumor growth in vivo and in vitro [81, 82]. In 2017, Yamashita et al reported that when 5-azacytidine was combined with temozolomide it increased survival in glioma models and further decreased tumor volumes [83]. Clinical trials evaluating DNMTi monotherapy with azacytidine (NCT03666559) and other DNMTi are investigating the role of these drugs for *mIDH* gliomas (NCT03922555).

### PARP Inhibitors and DNA Repair Enzymes

*IDH* mutations and subsequent accumulation of D-2-HG impair the integrity of homologous recombination-mediated double strand DNA break repair. This impairment forces *IDH* mutant glioma cells to use alternative repair mechanisms, such as those mediated by poly-ADP ribose polymerase (PARP) [78, 84].

PARP-mediated DNA repair is one of the mechanisms that remain intact in *IDH* mutant cells. These cells use this repair mechanism to maintain genomic integrity and survival during exposure to genotoxic therapy, such as radiation and chemotherapy [78, 85]. PARP inhibitors, such as olaparib, can induce a lethality to these cells by depriving them of this essential repair mechanism [86, 87].

A recent pre-clinical study reported that using PARP inhibitors in addition to radiation-induced DNA damage was highly effective in killing cells both in vivo and in vitro [88]. However, preliminary results from two clinical studies with olaparib in gliomas (NCT03561870 and NCT03212274) showed limited clinical benefit [89, 90]. Multiple clinical trials using PARP inhibitors as a single agent or in addition to temozolomide are ongoing (NCT03212742, NCT05297864, NCT03749187).

### Immunotherapies

*IDH* mutations are potential targets for immunotherapies as a tumor-specific neoantigen [91]. Moreover, D-2-HG induced DNA hypermethylation in gliomas results in silencing of programmed cell death-1 (PD-1) and its ligand (PDL-1) compared to *IDH* wild-type gliomas, which implies a stronger T-cell activation [92].

Multiple studies have reported on the suppression of the genes responsible for immune cell attraction in *IDH* mutant gliomas and the contributing role of D-2-HG as an inhibitor of anti-tumor immunity in the glioma microenvironment [93, 94]. In attempt to bypass this issue, IDH inhibitors have been added to checkpoint inhibitors and vaccine therapies to reduce the accumulation of D-2-HG and to subsequently to reverse the effect D-2-HG has on the immunity resulting in tumor volume reduction and prolonged OS [93, 94]. A recent preclinical study combined an IDH inhibitor with radiation and temozolomide in addition to a checkpoint inhibitor and reported improved survival in a mouse model [95]. Currently, multiple studies are looking at checkpoint inhibitors as monotherapy or in combination with other agents including IDH inhibitors, PARP inhibitors, or alkylating agents (NCT05188508, NCT04056910, NCT05484622).

Another immune-mediated strategy being investigated is using peptide vaccines that target the *IDH* neoantigen, which has shown positive results in mice [91, 96]. NOA-16 is a first-in-human, multicenter, phase I clinical trial using an *IDH1 R132H* peptide vaccine for *IDH* mutant high-grade astrocytomas. Recent results from this trial validate safety with no deaths, as well as vaccine-induced immunity in 93.3% of the patients enrolled and a three-year OS rate of 84% [97]. Two other peptide vaccines against *mIDH1* are being studied: PEPIDH1M in patients with recurrent grade 2 gliomas (RESIST trial NCT02193347) and *IDH1 R132H* dendritic cell vaccine in patients with *IDH* mutant gliomas (NCT02771301).

## Conclusion

The discovery of the *IDH* mutations represents a hallmark in the field of neuro-oncology, leading to significant progress in terms of glioma classification and prognosis and potential novel therapeutic approaches for these tumors. Guidelines for managing *IDH* mutant gliomas rely on studies conducted prior to the molecular era. These recommend surgical resection, radiation, and chemotherapy with variabilities in the timing of treatment and choice of chemotherapy between the different glioma grades and based on additional molecular alterations. While those guidelines still apply to current practice, the field is moving towards a more tailored approach in therapy strategies. Clinical researchers continue to seek new management methods that reflect the complexity and heterogeneity of these tumors while utilizing the advances made in understanding their behavior. Promising strategies continue to be developed, including targeting *IDH* mutations with *mIDH* inhibitors, focusing on DNA damage mechanisms with PARP inhibitors, and immunotherapies with checkpoint inhibitors, and peptide vaccines. Ongoing trials will help clarify the role of each of these therapies in the management of *mIDH* gliomas with a better understanding of their survival benefit as well as long-term toxicities. An examination of these strategies on a larger scale is needed to explore their benefit to the patients’ clinical course and overall survival in powered studies, and subsequently to influence a change in the existing guidelines.
